# Phase I/II Study of Stem-Cell Transplantation Using a Single Cord Blood Unit Expanded Ex Vivo With Nicotinamide

**DOI:** 10.1200/JCO.18.00053

**Published:** 2018-12-04

**Authors:** Mitchell E. Horwitz, Stephen Wease, Beth Blackwell, David Valcarcel, Francesco Frassoni, Jaap Jan Boelens, Stefan Nierkens, Madan Jagasia, John E. Wagner, Jurgen Kuball, Liang Piu Koh, Navneet S. Majhail, Patrick J. Stiff, Rabi Hanna, William Y.K. Hwang, Joanne Kurtzberg, Daniela Cilloni, Laurence S. Freedman, Pau Montesinos, Guillermo Sanz

**Affiliations:** ^1^ Duke University Medical Center, Durham, NC; ^2^ Emmes Corporation, Rockville, MD; ^3^ University Hospital Vall d’Hebron, Barcelona, Spain; ^4^ Istituto Giannina Gaslini, Genoa, Italy; ^5^ University Medical Center Utrecht, Utrecht, the Netherlands; ^6^ Vanderbilt University Medical Center, Nashville, TN; ^7^ University of Minnesota, Minneapolis, MN; ^8^ National University Health System, Singapore; ^9^ Cleveland Clinic, Cleveland, OH; ^10^ Loyola University Medical Center, Chicago, IL; ^11^ Singapore General Hospital, Singapore; ^12^ University of Turin, Turin, Italy; ^13^ Sheba Medical Center, Tel Hashomer, Israel; ^14^ Hospital Universitario y Politécnic de La Fe, Valencia, Spain

## Abstract

**Purpose:**

Increasing the number of hematopoietic stem and progenitor cells within an umbilical cord blood (UCB) graft shortens the time to hematopoietic recovery after UCB transplantation. In this study, we assessed the safety and efficacy of a UCB graft that was expanded ex vivo in the presence of nicotinamide and transplanted after myeloablative conditioning as a stand-alone hematopoietic stem-cell graft.

**Methods:**

Thirty-six patients with hematologic malignancies underwent transplantation at 11 sites.

**Results:**

The cumulative incidence of neutrophil engraftment at day 42 was 94%. Two patients experienced secondary graft failure attributable to viral infections. Hematopoietic recovery was compared with that observed in recipients of standard UCB transplantation as reported to the Center for International Blood and Marrow Transplant Research (n = 146). The median time to neutrophil recovery was 11.5 days (95% CI, 9 to 14 days) for recipients of nicotinamide-expanded UCB and 21 days (95% CI, 20 to 23 days) for the comparator (*P* < .001). The median time to platelet recovery was 34 days (95% CI, 32 to 42 days) and 46 days (95% CI, 42 to 50 days) for the expanded and the comparator cohorts, respectively (*P* < .001). The cumulative incidence of grade 2 to 4 acute graft-versus-host disease (GVHD) at day 100 was 44%, and grade 3 and 4 acute GVHD at day 100 was 11%. The cumulative incidence at 2 years of all chronic GVHD was 40%, and moderate/severe chronic GVHD was 10%. The 2-year cumulative incidences of nonrelapse mortality and relapse were 24% and 33%, respectively. The 2-year probabilities of overall and disease-free survival were 51% and 43%, respectively.

**Conclusion:**

UCB expanded ex vivo with nicotinamide shortens median neutrophil recovery by 9.5 days (95% CI, 7 to 12 days) and median platelet recovery by 12 days (95% CI, 3 to 16.5 days). This trial establishes feasibility, safety, and efficacy of an ex vivo expanded UCB unit as a stand-alone graft.

## INTRODUCTION

Despite remarkable improvement in outcomes of adult recipients of umbilical cord blood (UCB) transplantation, slow hematopoietic recovery continues to be the major limitation of this approach. Stemming from this delay in hematopoietic recovery are other disadvantages of UCB transplantation, such as increased risk for infection, prolonged hospitalization, and increased resource use. Early-phase, single-center studies have demonstrated that ex vivo expansion of UCB stem cells before transplantation has the potential to address this critical shortcoming. By expanding both hematopoietic stem and progenitor cells, the time to neutrophil recovery after myeloablative conditioning can be even more rapid than that after a mobilized peripheral blood stem-cell graft.^1-4^

NiCord (Gamida Cell, Jerusalem, Israel) is an ex vivo expanded cell product derived from the CD133+ fraction of banked UCB that uses nicotinamide as the active agent that inhibits differentiation and enhances the functionality of cultured hematopoietic stem and progenitor cells. When nicotinamide is added to stimulatory hematopoietic cytokines, UCB-derived hematopoietic progenitor cell cultures demonstrate an increased frequency of phenotypically primitive CD34^+^CD38^−^ cells and a substantial increase in bone marrow homing and engraftment potential of ex vivo expanded CD34+ cells.^5^ The ability of nicotinamide to expand both committed and long-term repopulating hematopoietic stem cells was confirmed in a first-in-human pilot study of NiCord.^3^ In this study, a second unmanipulated UCB unit was coinfused with the NiCord expanded unit to maintain patient safety. With long-term follow-up, stable NiCord-derived hematopoiesis has now been observed for more than 7 years. On the basis of these results, we conducted a multicenter, phase I/II study of NiCord transplanted as a single, expanded UCB graft after myeloablative conditioning.

## METHODS

### Patient Eligibility

Eligible patients were 12 to 65 years of age with high-risk hematologic malignancies and no readily available matched sibling or matched unrelated adult donor. The Center for International Blood and Marrow Transplant Research (CIBMTR) provided historical data on 1,037 patients undergoing UCB transplantation between 2010 and 2013. A cohort of patients was selected with characteristics as similar as possible to the phase I/II patients; selections for myeloablative conditioning, disease status, age, graft size, HLA matching, and performance score criteria resulted in a CIBMTR sample size of 146 (Appendix Table A1, online only). Among the final cohort, 80% received a double cord blood graft and 20% received a single cord blood graft.

**FIG A1.**
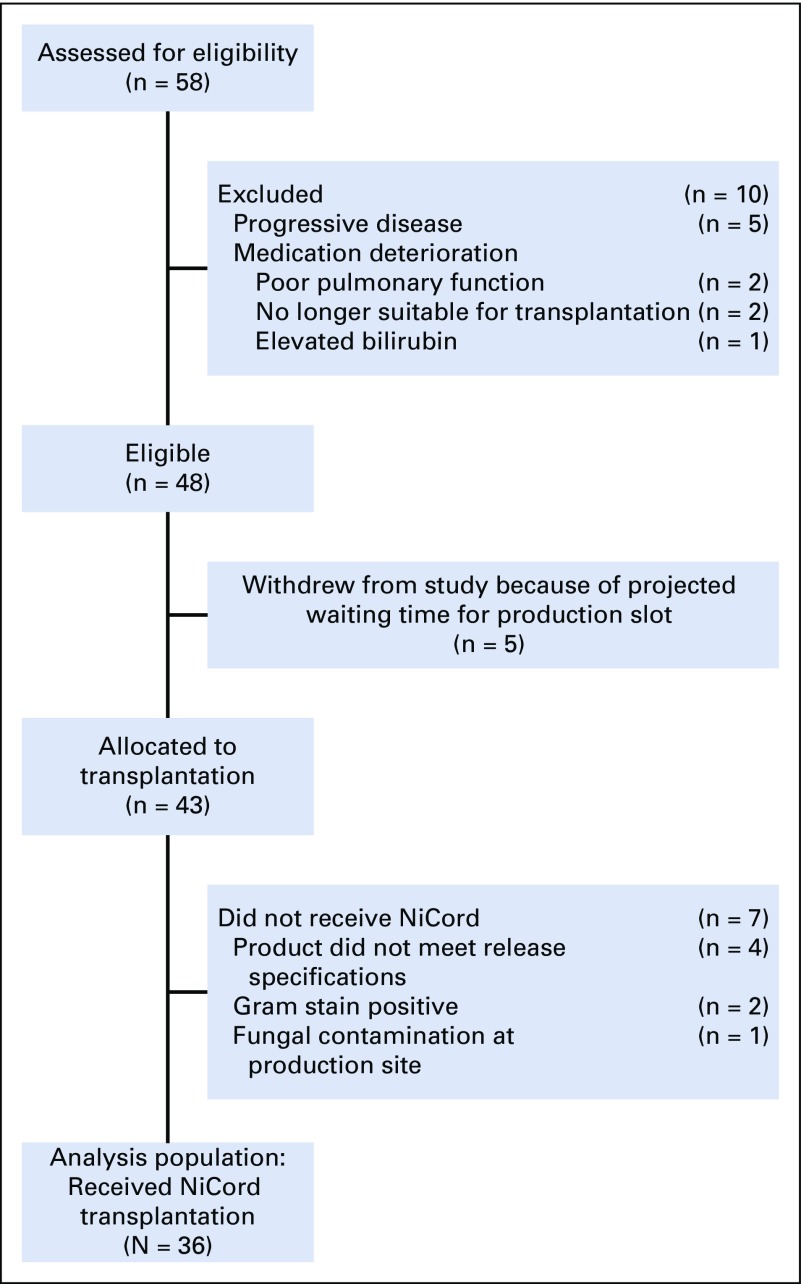
CONSORT diagram.

Of the 58 patients who enrolled in the trial between 2013 and 2017, 10 became ineligible during the pretransplantation work-up and five withdrew because of logistical issues surrounding graft production. Forty-three patients were allocated to treatment in the study. Seven of the 43 patients were not evaluable because of NiCord production complications. These patients underwent UCB transplantation with either an unmanipulated cord blood graft or a combination of NiCord plus an unmanipulated cord blood graft (Appendix Fig A1, online only).

The study was approved by the institutional review boards of all participating institutions and the national regulatory authorities. All patients provided written informed consent. The study was performed in accordance with the International Conference on Harmonization Guidelines and Good Clinical Practice (ClinicalTrials.gov identifier: NCT01816230).

### Graft Selection

Protocol eligibility required patients to have a cord blood unit matched at 4 to 6/6 HLA class I (HLA-A and HLA-B, low resolution) and class II (HLA-DRB1, high resolution) loci (Data Supplement). The unit was required to have a precryopreserved dose greater than or equal to 8.0 × 10^6^ CD34+ total cells as well as a precryopreserved total nucleated cell dose (TNC) greater than or equal to 1.8 × 10^9^ delivering greater than or equal to 1.8 × 10^7^ TNC/kg. The UCB unit must have been volume reduced and red blood cell depleted before cryopreservation. UCB bank preference was not specified in the eligibility criteria. An additional partially HLA-matched cord blood unit of at least 2.5 × 10^7^ TNC/kg was reserved as a backup in case the expanded product did not pass the required quality control tests.

### NiCord Production

The NiCord-designated unit was delivered from the cord blood bank to a Current Good Manufacturing Practice–compliant cell-processing facility (Lonza, MD, or Gamida Cell, Jerusalem, Israel). NiCord was manufactured as previously described.^3^ Briefly, the unit underwent immunomagnetic bead selection for CD133+ cells. The CD133−, T-cell–containing flow-through fraction was retained and recryopreserved. The CD133+ fraction was cultured for 21 ± 2 days and then recryopreserved. Taking into account time for shipment of the UCB unit to the cell-processing facility and then to the transplant center, the total time for NiCord production is 24 ± 3 days. CD34+ and CD3+ cell content of the graft, as reported in this article, was quantified before recryopreservation of the product.

### Conditioning Regimens and Graft-Versus-Host Disease Prophylaxis

Three alternative myeloablative conditioning regimens were permitted for study participants (Table 1). All dosing was on the basis of 25% adjusted ideal body weight unless otherwise noted.

**TABLE 1.**
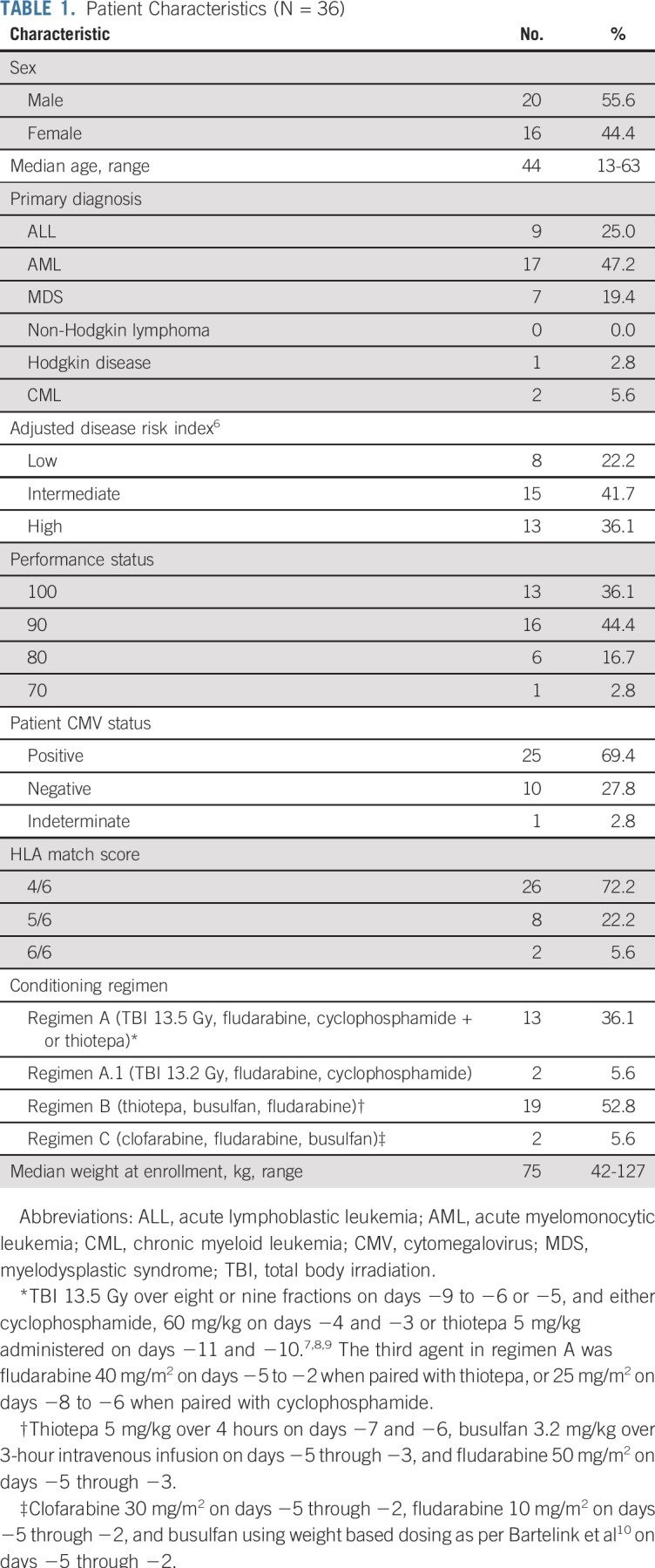
Patient Characteristics (N = 36)

Graft-versus-host disease (GVHD) prophylaxis was provided by a calcineurin inhibitor (tacrolimus or cyclosporine) and mycophenolate mofetil starting 4 days before transplantation. Mycophenolate mofetil was continued for a minimum of 60 days and the calcineurin inhibitor for minimum of 6 months after transplantation.

### Supportive Care

Granulocyte colony-stimulating factor (5 µg/kg recipient body weight) was administered daily starting on day +1 after transplantation until the absolute neutrophil count exceeded 1,000 cells/µl. Antiviral and antifungal prophylaxis were administered at the discretion of the transplantation center. Antibacterial prophylaxis for the first 100 days after transplantation was required by protocol. The agent used was left to the discretion of the transplant center.

### Laboratory and Clinical Assessments

Donor chimerism was performed by the local transplant center on whole blood, CD15+ myeloid, and CD3+ T cells using quantitative analysis of informative microsatellite DNA sequences. Quantitative assessment of CD3, CD4, CD8, natural killer, and B-cell recovery was performed on a subset of patients by the local transplantation center (or designated referral laboratory) 2 months, 3 months, 6 months, and 1 year after transplantation. The time to neutrophil and platelet engraftment was defined as per CIBMTR standards.

### Statistical Considerations

Analysis was limited to the 36 patients undergoing transplantation with NiCord as a stand-alone graft. Database closure was on November 16, 2017.

The primary end points were the cumulative incidence of neutrophil engraftment at 42 days with less than or equal to 10% host cells and the incidence of secondary graft failure. To facilitate comparison with CIBMTR data, engraftment without chimerism was evaluated here. Secondary end points evaluated here were cumulative incidence of platelet engraftment, overall survival, nonrelapse mortality, disease relapse, acute and chronic GVHD, and time alive and out of hospital over the first 100 days. Competing risks for engraftment were death, progression/relapse, and second transplantation; for GVHD, competing risks were death, absolute neutrophil count recovery failure, second transplantation, secondary graft failure, and progression/relapse; for nonrelapse mortality, competing risk was progression/relapse; and for progression/relapse, competing risk was death.

Because of differences in the age distribution between the phase I/II study and the retrospective cohort, unadjusted and age-adjusted cumulative incidence curves for engraftment were calculated; age-adjusted curves for the CIBMTR cohort were weighted by the proportion of patients in the phase I/II trial in age strata 18 years or younger, 19 to 39 years, and 40 years or older. Comparison graphs for engraftment are provided showing the weighted cumulative incidence. Calculations of SEs for the unadjusted and adjusted cumulative incidence CIs were based on the Aalen and delta method, respectively. Differences between times to engraftment were tested using a van Elteren test stratified on age groups. For these tests, patients not engrafting were assigned a time to event larger than any patient with an event. Median time to an event is calculated among those with an event, with 95% CIs on the basis of CI calculations for rank statistics. CIs (95%) for the difference between median times to engraftment were estimated using the nonparametric bootstrap. For secondary end points, unadjusted cumulative incidence or Kaplan-Meier survival probabilities are reported. For GVHD, comparisons were made using the Fine-Gray model with group and age group as covariates; for nonrelapse mortality and relapse, comparisons were made using the Fine-Gray model with group, age group, and disease risk index, and Cox models were also used to help interpret the results. For overall survival and disease-free survival, comparisons were made using the log-rank test and a Cox model with group, age group, and disease risk index. Time out of hospital was compared using the age-stratified van Elteren test. SAS (SAS/STAT User’s Guide, Version 9.4, SAS Institute, Cary, NC), STATA 15 software (STATA, College Station, TX; Computing Resource Center, Santa Monica, CA), R Studio, and R3.3.1 or higher were used for these analyses.

## RESULTS

### Patient and Stem-Cell Transplantation Characteristics

Patient characteristics are described in Table 1. Eleven centers in the United States, Europe, and Asia (Singapore) enrolled patients on the study.

### Graft Characteristics

**FIG 1.**
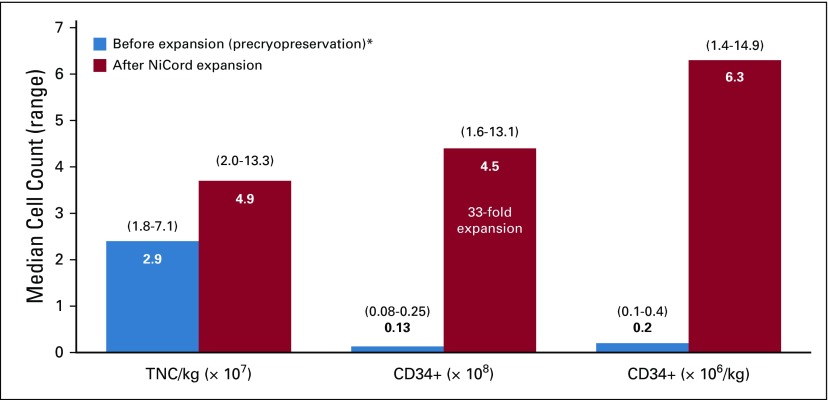
NiCord graft characteristics. Median (range) total nucleated cell (TNC) content, median (range) CD34+ cell content, and median (range) CD34+ cell dose are shown before and after ex vivo expansion of the umbilical cord blood unit. (*) Pre-expansion values represent cell content as reported by the cord blood bank before cryopreservation of the umbilical cord blood unit.

Characteristics of the NiCord graft before and after expansion are shown in Figure 1. The median total CD34+ cell content of the cord blood unit as reported by the cord blood bank before cryopreservation and expansion was 0.13 × 10^8^ (range, 0.08 to 0.25 × 10^8^). After NiCord expansion, the CD34 content of the graft increased by 33-fold to a median 4.5 × 10^8^ (range, 1.6 to 13.1 × 10^8^) CD34+ cells. This resulted in a median CD34+ cell dose of 6.3 × 10^6^/kg (range, 1.4 to 14.9 × 10^6^/kg). The CD3+ T cells in the NiCord graft were contained solely in the unexpanded, CD133-negative fraction. CD3+ T-cell content of the negative fraction was a median of 2.0 × 10^8^ (range, 0.7 to 14.0 × 10^8^), resulting in a median CD3+ content of 2.4 × 10^6^/kg (range, 0.7 to 24.0 × 10^6^/kg).

### Hematopoietic Recovery

**FIG 2.**
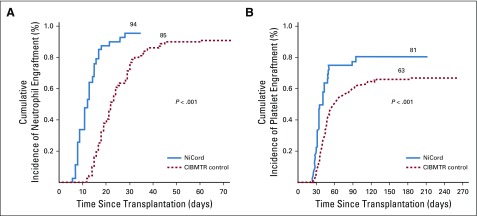
Hematopoietic recovery. (A) Weighted cumulative incidence of neutrophils by day 42, and (B) platelet recovery by day 100 among recipients of NiCord and a comparable retrospective cohort from the Center for International Blood and Marrow Transplant Research.

The age-adjusted cumulative incidence of neutrophil engraftment at 42 days after transplantation was 94% for NiCord recipients and 85% for the CIBTMR comparator cohort (Fig 2A). By 21 days after transplantation, 89% of NiCord recipients had achieved neutrophil engraftment. Neutrophil engraftment was faster for NiCord recipients (*P* < .001). Among patients who engrafted, the median time to neutrophil recovery was 11.5 days (95% CI, 9 to 14 days) for NiCord recipients and 21 days (95% CI, 20 to 23 days) for the CIBMTR comparator cohort. The age-adjusted cumulative incidence of platelet engraftment at 100 days after transplantation was 81% for NiCord recipients and 63% for CIBMTR comparator cohort (Fig 2B). Platelet engraftment was faster among NiCord recipients (*P* < .001). For patients who achieved platelet recovery, the median time to platelet recovery was 34 days (95% CI, 32 to 42 days) and 46 days (95% CI, 42 to 50 days) for NiCord and CIBMTR comparator cohorts, respectively.

Whole blood chimerism was available for 26 patients at 100 days after transplantation. Twenty-five patients (96%) had greater than or equal to 95% and one had 57% donor whole blood chimerism. Lineage-specific myeloid and T-cell chimerism was available in a subset of patients (n = 22) at day 100. Twenty patients had greater than 90% donor chimerism in both fractions. Two patients had mixed chimerism at day 100; one was 57% in the myeloid fraction and 3% in the T-cell fraction and the other was 100% in the myeloid fraction and 10% in the T-cell fraction.

One patient experienced primary graft failure. Two patients experienced secondary graft failure, one occurring at day 19, concurrent with high titer human herpes virus 6 viremia, and the second occurring at day 262, concurrent with a lethal adenovirus infection.

### GVHD

The unadjusted cumulative incidence of grade 2 to 4 acute GVHD at day 100 was 44% (95% CI, 28% to 60%) for the NiCord recipients and 56% (95% CI, 47% to 64%**)** for the CIBMTR comparator cohort, and the Fine-Gray model hazard ratio (HR) was nonsignificant (HR, 0.7; *P* = .20). The unadjusted cumulative incidence of grade 3 or 4 acute GVHD at day 100 was 11% (95% CI, 3% to 24%) for the NiCord recipients and 27% (95% CI, 20% to 34%**)** for the CIBMTR comparator cohort, and the Fine-Gray HR was nonsignificant (HR, 0.4; *P* = .09). The unadjusted cumulative incidence of chronic GVHD at 2 years was 40% (95% CI, 24% to 57%) for NiCord recipients and 30% (95% CI, 22% to 37%) for the CIBMTR comparator cohort, and the Fine-Gray model HR was nonsignificant (HR, 1.6; *P* = .1). The 2-year cumulative incidence of moderate to severe chronic GVHD was 10% (95% CI, 2% to 24%) for NiCord recipients and 10% (95% CI, 6% to 16%) for the CIBMTR comparator cohort.

### Nonrelapse Mortality, Relapse, Disease-Free Survival, and Overall Survival

**FIG 3.**
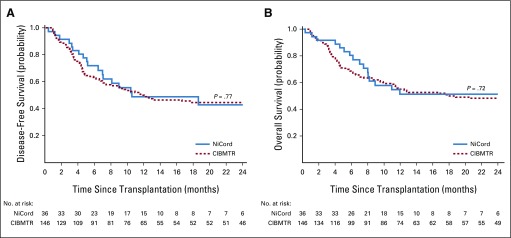
Kaplan-Meier estimate and log-rank test of (A) disease-free survival and (B) overall survival after transplantation with NiCord and a comparable retrospective cohort from the Center for International Blood and Marrow Transplant Research (CIBMTR).

The median follow-up of surviving NiCord recipients was 14 months (range, 5 to 36 months). The unadjusted 2-year cumulative incidence of nonrelapse mortality for NiCord recipients was 24% (95% CI, 11% to 39%). Using both the Fine-Gray and the Cox models, 2-year nonrelapse mortality hazard rates were lower for patients receiving a NiCord graft compared with the CIBMTR cohort (Table 2). The unadjusted 2-year cumulative incidence of relapse for NiCord recipients was 33% (95% CI, 16% to 52%). Cause-specific hazard for relapse for NiCord recipients was no different from the CIBMTR cohort when compared using the Cox model, but the subdistribution hazard was higher when compared using the Fine-Gray model (Table 2). The 2-year probability of disease-free survival for NiCord recipients was 43% (95% CI, 24% to 60%) and 45% (95% CI, 37% to 53%) for the CIBMTR comparator cohort (*P* = .77). The unadjusted 2-year probability of overall survival for NiCord recipients was 51% (95% CI, 33% to 67%) and 48% (95% CI, 40% to 56%) for the CIBMTR comparator cohort (*P* = .72; Fig 3). With adjustment for both age and disease-risk index, there were no differences in disease-free and overall survival hazard between the two cohorts (Table 2).

**TABLE 2.**
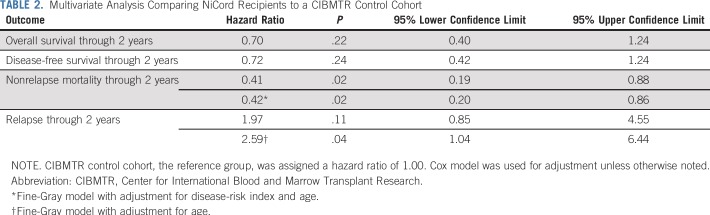
Multivariate Analysis Comparing NiCord Recipients to a CIBMTR Control Cohort

### Transplantation Course and Toxicity

Primary hospital discharge occurred at a median of 20 days (range, 0 to 61 days) after transplantation. Recipients of the NiCord graft spent a median of 73 days (range, 0 to 85 days), and CIBMTR standard cord blood recipients spent a median of 57 days (n = 141; range, 0 to 92 days) alive and out of the hospital during the first 100 days after UCB transplantation (*P* < .001). Hypertension was reported as the most common toxicity attributable to NiCord infusion. One grade 3 hypertension and one grade 2 hypersensitivity reaction were attributed to NiCord infusion. Of the 16 patients who died, eight deaths (50%) were attributable to relapsed disease, five (31%) to infection, two (13%) to GVHD, and one (6%) to organ failure.

### Immune Reconstitution

**FIG 4.**
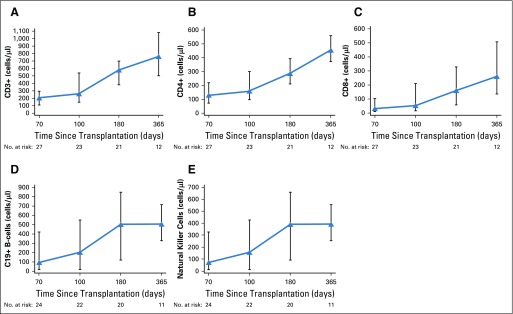
Immune reconstitution. Median and interquartile range for quantitative recovery of (A) CD3, (B) CD4, (C) CD8, (D) CD19, and (E) natural killer cells measured at target day 70, day 100, 6 months, and 1 year after transplantation with NiCord.

Lymphoid immune recovery was monitored in a subset of 27 patients after transplantation of NiCord. Figure 4 demonstrates the CD3, CD4, CD8, CD19, and natural killer cell recovery during the first 12 months after transplantation.

## DISCUSSION

NiCord is an ex vivo expanded UCB graft designed specifically to address the limitations arising from low hematopoietic stem and progenitor cell dose and resultant delayed engraftment after adult UCB transplantation. We show that transplantation of NiCord is safe, is effective in reducing the time to hematopoietic recovery, and does not require coinfusion of a second unmanipulated UCB unit.

The use of dual UCB grafts has vastly expanded the accessibility of UCB transplantation to adult patients who lack an adequately sized single UCB graft.^7,8^ However, the problem of delayed hematopoietic recovery was not addressed by this technique. Ex vivo expansion of UCB stem and progenitor cells has been studied by a number of groups in an attempt to address the important limitation of UCB transplantation.^1,2,4,11,12^ Delaney and colleagues^1^ were the first to demonstrate that transplantation of UCB stem cells, expanded in the presence of Delta 1 Notch ligand, resulted in a median 10-day reduction in time to neutrophil recovery compared with conventional dual UCB transplantation. This strategy was designed as a bridge to long-term engraftment by a second, unmanipulated UCB graft.

NiCord was designed to be a stand-alone graft and differed from the preceding expanded UCB products in that the T-cell fraction from the unit was retained and recryopreserved before culture. This important difference allowed NiCord the potential to become the dominant unit after coinfusion with an unmanipulated cord blood unit.^3^

To our knowledge, this study is the first to show that an expanded UCB unit can be infused as a stand-alone graft and is capable of providing robust, durable hematopoiesis. One patient (3%) experienced primary graft failure, a rate well below the graft failure rate after stem-cell transplantation from bone marrow grafts.^13^ Two patients experienced secondary graft failure. Although stem-cell exhaustion cannot be completely ruled out, high titer adenovirus and human herpesvirus 6 infections are the most plausible explanation for these events.

The median time to neutrophil recovery is 20 days after myeloablative HLA-identical allogeneic bone marrow transplantation and 15 days after HLA-identical mobilized peripheral blood stem-cell transplantation.^13^ This 5-day reduction in time to neutrophil recovery for peripheral blood stem-cell recipients translated into a significant reduction of bacterial infections during the first 100 days after transplantation.^14^ The median time to neutrophil recovery after myeloablative haploidentical peripheral blood stem-cell transplantation using post-transplantation cyclophosphamide as GVHD prophylaxis is 16 to 19 days.^15,16^ Transplantation of NiCord as a single ex vivo expanded UCB graft results in an estimated median time to neutrophil recovery of 11.5 days (95% CI, 9 to 14 days). This marked reduction in time to neutrophil recovery explains why NiCord recipients spent less time in the hospital compared with the CIBMTR cohort and why the NiCord graft has been associated with a reduction in bacterial infections.^17^

When compared with a retrospective cohort of patients who received standard myeloablative UCB transplantation, we observed that recipients of NiCord experienced a trend toward less severe acute GVHD, lower nonrelapse mortality, and higher relapse. The lower nonrelapse mortality in the NiCord cohort could confound the comparison of relapse. Results using the Cox instead of the Fine-Gray model indicated a smaller, statistically nonsignificant (*P* = .11), increase in the relapse rate among NiCord recipients. Overall, these findings need to be considered with caution. The small sample size of the NiCord cohort resulted in wide confidence intervals. Extreme disease heterogeneity among the cohorts could also confound the comparison of relapse.

UCB transplantation has a 30-year track record of providing a hematopoietic stem-cell transplant option for patients without an available matched adult donor.^18^ Many adult recipients require two UCB units to ensure reliable engraftment. However, the addition of a second unit significantly increases the expense of the transplantation and is associated with delayed platelet recovery and a higher incidence of chronic GVHD.^19^ This study suggests that NiCord obviates the need for a second UCB graft. NiCord paves the way for use of smaller, better-matched units for adult patients that otherwise could not be used because of excessive risk of graft failure. Additional studies will be needed to determine whether the time required for graft production negatively affects patient outcome. The study demonstrates the feasibility of an ex vivo expanded hematopoietic stem-cell product manufactured in a centralized cell-processing facility and distributed internationally to three continents. It is hypothesized that an ongoing prospective, multicenter, phase III registration trial comparing NiCord to standard myeloablative UCB transplantation will provide confirmation of the findings presented in this study.

## Figures and Tables

**TABLE A1.**
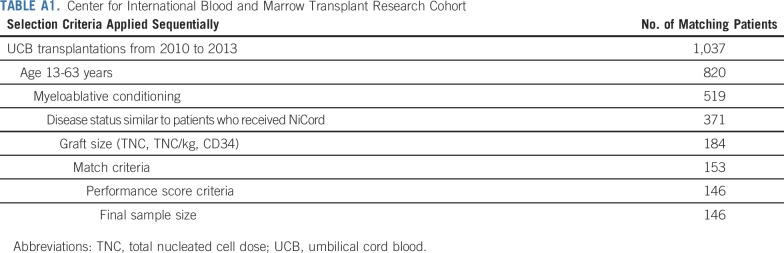
Center for International Blood and Marrow Transplant Research Cohort
